# ALG-2 Attenuates COPII Budding In Vitro and Stabilizes the Sec23/Sec31A Complex

**DOI:** 10.1371/journal.pone.0075309

**Published:** 2013-09-19

**Authors:** Jonas M. la Cour, Adam J. Schindler, Martin W. Berchtold, Randy Schekman

**Affiliations:** 1 From the Department of Biology, University of Copenhagen, Copenhagen, Denmark; 2 From the Biology Department, Duke University, Durham, North Carolina, United States of America; 3 From the Department of Cellular Biochemistry, University of California at Berkeley, Berkeley, California, United States of America; Tohoku University, Japan

## Abstract

Coated vesicles mediate the traffic of secretory and membrane cargo proteins from the endoplasmic reticulum (ER) to the Golgi apparatus. The coat protein complex (COPII) involved in vesicle budding is constituted by a GTPase, Sar1, the inner coat components of Sec23/Sec24 and the components of the outer coat Sec13/Sec31A. The Ca^2+^-binding protein ALG-2 was recently identified as a Sec31A binding partner and a possible link to Ca^2+^ regulation of COPII vesicle budding. Here we show that ALG-2/Ca^2+^ is capable of attenuating vesicle budding in vitro through interaction with an ALG-2 binding domain in the proline rich region of Sec31A. Binding of ALG-2 to Sec31A and inhibition of COPII vesicle budding is furthermore dependent on an intact Ca^2+^-binding site at EF-hand 1 of ALG-2. ALG-2 increased recruitment of COPII proteins Sec23/24 and Sec13/31A to artificial liposomes and was capable of mediating binding of Sec13/31A to Sec23. These results introduce a regulatory role for ALG-2/Ca^2+^ in COPII tethering and vesicle budding.

## Introduction

Ca^2+^ is a ubiquitous messenger molecule regulating a wide array of cell biological processes. Recent evidence suggests that Ca^2+^ transients may also be involved in regulatory mechanisms related to protein trafficking. The Ca^2+^ binding protein ALG-2 (product of the apoptosis linked gene-2) was found to interact with Sec31A, a component of the coat protein complex II (COPII) in a Ca^2+^-dependent manner. It has thus been speculated that ALG-2 mediates a Ca^2+^-link to COPII dependent protein traffic [1], [2], [3]. During physiological Ca^2+^ transients a major part of the cytosolic ALG-2 redistributes to cytoplasmic puncta [1] where it colocalizes with Sec31A [1], [2], [3] and the ER exit site marker p125 [3]. Ca^2+^ signaling has been shown to be mandatory for some aspects of vesicle trafficking such as synaptotagmin-mediated membrane fusion [4]. Furthermore, recent work by Bentley et al. suggests that Ca^2+^ together with ALG-2 plays a regulatory role in the fusion of COPII vesicles after budding [5]. ALG-2 was originally assigned a functional role in apoptosis linking Ca^2+^ signaling to programmed cell death [6], however, no molecular mechanism underlying this function has been described. In addition, the suggested apoptotic function of ALG-2 was not confirmed by gene knock out studies in mice [7].

Protein export from the ER is facilitated by vesicles encaged by the Sar1, Sec23–Sec24 and Sec13–Sec31 proteins constituting a COPII vesicle (reviewed in [8]) Defects in COPII component expression were found to lead to skeletal abnormalities [9] and improper extracellular matrix formation [10]. Structural data suggests a COPII cage diameter size of 60–80 nm [11], contrasting with findings of COPII-dependent export of collagen [12], which would require larger vesicles (reviewed in [13]). This has led to speculation that a specialized mechanism to accommodate bulky cargo is needed, and evidence supporting this has come with the recent discovery of a role for ubiquitin conjugation of Sec31 in the formation of enlarged COPII- vesicles facilitating collagen export [14]. How the COPII structures are retained at the ER exit sites in order to expand to a size large enough to accommodate bulk cargo remains unexplained. Here we show that Ca^2+^-ALG-2 attenuates the exit of protein from the ER through an interaction with the proline rich region of Sec31A. Furthermore, we find that ALG-2 in vitro is capable of bridging the outer and inner components, Sec31A and Sec23 of COPII.

## Results

### ALG-2 Inhibits in vitro Budding in a Ca^2+^-dependent Manner

**Figure 1:**
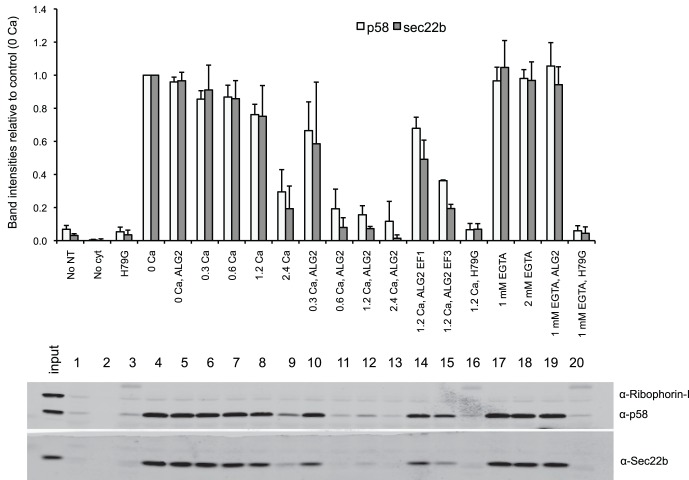
ALG-2 attenuates COPII dependent budding. The budding efficiency was measured from the amount of p58 and Sec22b in vesicles relative to the amount in donor membranes. HeLa cell donor membranes were incubated with rat liver cytosol, ATP and an ATP regenerating system, and wild-type recombinant ALG-2 (lanes 10–13) or ALG-2 with mutant versions of either EF-hand 1 (ALG2EF1, lane 14) or EF-hand 3 (ALG2EF3, lane 15) in the presence of either Ca^2+^ (lanes 6–16) or chelator (EGTA, lanes 17–20). As controls, membranes were incubated in the absence of either nucleotides (No NT, lane 1) or cytosol (No cyt, lane 2). As a control for the requirement of COPII proteins, we performed assays in the presence of a GTP-restricted dominant-negative form of the GTPase Sar1, Sar1 (H79G, lanes 3, 16 and 20). Budding efficiency was measured from band intensities and normalized to the amount of budding in the no Ca^2+^, no ALG-2 vesicle budding (0 Ca, lane 4) reaction. Bars indicate average +/− SD of three independent experiments.

ALG-2 was previously described to interact with Sec31A in a Ca^2+^-dependent manner [1], [2], [3]. However, the significance of this interaction for ER to Golgi protein transport is not seen in the trafficking of VSV-G in cells depleted of ALG-2 [3]. In order to test the effect of ALG-2 on COPII budding under controlled Ca^2+^-conditions, we investigated the consequences of adding recombinant ALG-2 to a cell-free vesicle budding reaction in the presence or absence of free Ca^2+^. Using two transit cargo marker proteins, the SNARE protein Sec22b and the ER-Golgi cargo protein p58/ERGIC, we were able to measure the efficiency of COPII-dependent vesicle budding. Increasing Ca^2+^ concentrations (Figure 1, data points 4–9) correlated with decreased budding efficiency, which was further decreased in the presence of recombinant ALG-2 (Figure 1, data points 10–13). Ca^2+^ inhibited budding starting at concentrations greater than 1.2 mM, whereas addition of recombinant ALG-2 inhibited budding at a lower concentration of 300 uM Ca^2+^. Non-physiological concentrations of Ca^2+^ were used to override the Ca^2+^ chelator EGTA from the rat liver cytosol preparations. Mutant versions of ALG-2 that are partially deficient in Ca^2+^-binding led to a partial recovery of the budding efficiency at elevated Ca^2+^ concentration most prominently when EF hand 1 was inactivated by mutation (Figure 1, data points 14 and 15). The inhibitory effect of wild-type ALG-2 was abolished when further EGTA was added (Figure 1, data points 17 and 19), demonstrating that ALG-2 requires Ca^2+^ for its effect on budding. In all conditions, budding was sharply reduced in incubations containing a dominant negative form of the COPII protein Sar1p (Sar1H79G, Figure 1, data points 3, 16 and 20), showing the requirement for functional COPII in the budding reaction.

### ALG-2 Acts Directly on COPII to Inhibit Vesicle Budding in a Reversible Manner

**Figure 2:**
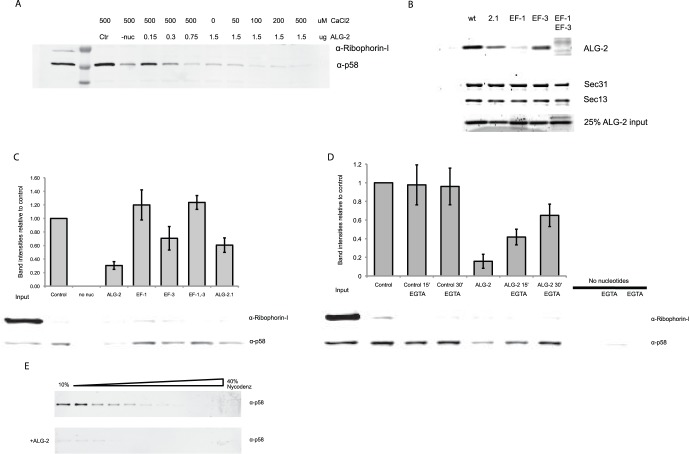
ALG-2 attenuates in vitro reconstituted COPII vesicle budding in a reversible manner. Budding investigated using p58/ERGIC as a marker after incubation of HeLa cell donor membranes with recombinant Sar1A, Sec23A/24D, Sec13/31A and 0–2 ug of recombinant ALG-2. **A.** Budding efficiency was measured at increasing concentrations of either ALG-2 or Ca^2+^. **B.** Attenuation of budding was dependent on EF hand 1 of ALG-2. Binding of ALG-2 wildtype (wt) and mutants to Sec31A was tested by incubating either wt ALG-2 (wt), ALG-2.1 (2.1), ALG-2mutEF1 (EF-1), ALG-2mutEF3 (EF-3) or ALG-2mutEF1&3 (EF-1–3) with Sec31A-coated beads. Eluates were separated by SDS-PAGE and visualized using SyproRed. **C.** *In vitro* reconstituted budding using p58 as a budding marker. Mutant versions of ALG-2 were added to the budding reaction in equimolar amounts. D. ALG-2/Ca^2+^ budding attenuation was reversed by chelation of Ca^2+^. In vitro reconstituted vesicle budding using p58 as a cargo protein marker. Budding was performed for 45 min in the presence of Ca^2+^ only (control), Ca^2+^/ALG-2 (ALG-2) or in absence of nucleotides. EGTA was added to budding reactions at T = 15 and T = 30 min and reactions were incubated in the presence of 1 mM EGTA for the indicated time periods (min). Background budding levels were found by omitting nucleotides (no nuc). SEM of three independent experiments. E. 10–40% Nycodenz gradient was layered on top of the product of the budding reactions performed in the absence (upper panel) or presence (lower panel) of recombinant ALG-2.

To avoid the presence of chelator and auxiliary factors present in the liver cytosol that could interfere with the ALG-2/Ca^2+^ effect on budding (Figure 1), we reconstituted the budding assay with pure recombinant Sar1A, Sec23A/24D, and Sec13/31A in place of cytosol [15]. We found that ALG-2/Ca^2+^ acted directly through COPII and using various concentrations of both ALG-2 and Ca^2+^ found that budding was inhibited when the molar ratio of ALG-2 to Sec31A exceeded 2∶1 (Figure 2A). In this reconstitution, budding was inhibited by ALG-2 even when no Ca^2+^ was added, indicating that in the absence of chelator, background Ca^2+^ levels from the donor membrane preparations are sufficient to inhibit budding in concert with ALG-2 (Figure 2A). It was previously shown that ALG-2 redistribution from the cytosol to ER exit site structures is observed following induction of Ca^2+^ transients with histamine or other stimuli [1]. Ca^2+^ binds to the high affinity EF hands 1 and 3 of ALG-2 with a K_D_ of 1.3 µM [16], which is within the range of the Ca^2+^-levels reached during cellular Ca^2+^ transients evoked by histamine [17]. To ensure that free Ca^2+^ was available for activation of ALG-2, we added 10 µM Ca^2+^ to the in vitro reconstituted budding assay unless otherwise stated. To investigate the partial inhibitory effect of the ALG-2 EF hand mutants in budding, we performed binding studies by incubating recombinant ALG-2 versions with His-tagged Sec31A immobilized on Nickel-beads. In the presence of Ca^2+^, only the splice variant of ALG-2, ALG-2.1 [16], and ALG-2 with the E114A substitution in EF-hand 3 were able to bind to Sec31A (Figure 2B), indicating that EF1-hand 1 is mandatory for this interaction. Furthermore, using the ALG-2 mutants in the budding assay, we showed that the binding capacity of the ALG-2 versions correlated with their ability to inhibit budding in a Ca^2+^-dependent manner (Figure 2C). This indicates that an EF-hand1-dependent binding of ALG-2 to Sec31A is associated with the Ca^2+^-mediated inhibition of vesicle budding. To support the proposed correlation between the binding of ALG-2 and Sec31A with budding inhibition and the Ca^2+^-dependent oscillatory pattern of ALG-2 distribution in the microscopy experiments, we performed the budding reaction in the presence of exogenous Ca^2+^. EGTA was added after 15 and 30 min to remove Ca^2+^ in order to allow disruption of the ALG-2/Sec31A complex. Following addition of EGTA, the budding efficiency was restored in the reactions where ALG-2 was present (Figure 2D), suggesting that Ca^2+^ chelation abolishes the ALG-2 attenuation of COPII vesicle budding. To identify possible changes in vesicular budding products as a consequence of adding ALG-2, we resolved membranes in the budding reaction on a Nycodenz buoyant density gradient and fractions were analyzed for the presence of the budding marker p58/ERGIC. In the presence of ALG-2, only trace amounts of p58/ERGIC were found as compared to control conditions in which p58/ERGIC was found in light vesicle fractions (Figure 2E). The resident ER marker protein ribophorin I, which is retained in the ER, was undetectable in the fractions (not shown). These findings indicate that vesicles containing marker cargo do not bud from the ER in the presence of ALG-2/Ca^2+^.

### The Inhibitory Effect of ALG-2 on Budding is Mediated through Sec31A

**Figure 3:**
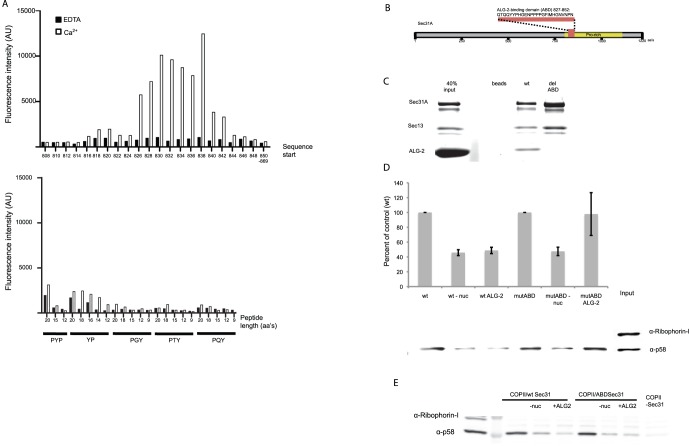
Binding of ALG-2 to Sec31A attenuates COPII budding. **A.** Peptide array mapping of the ALG-2-binding domain (ABD) of Sec31A. Binding efficiency of fluorescein tagged recombinant ALG-2 binding to 20-mer peptides (upper panel). Various lengths of the indicated proline rich repeats were used as controls (lower panel) **B.** Diagram representing the ABD of Sec31A and the sequence removed from the Sec31aAdelABD. **C.** Recombinant ALG-2 was incubated with Ni-beads alone, with purified His-tagged Sec13/Sec31A (wt) or Sec13/Sec31A with the ABD deleted (delABD) in the presence of Ca^2+^. Proteins in the gel were visualized using SyproRed. **D.** Budding was performed using purified Sar1A, Sec23A/24D and either wt Sec13/31A (wt) or Sec13/31A with the ABD of Sec31A deleted (delABD) in the presence of 0.15 ug ALG-2. Background budding levels were found by omitting nucleotides (-nuc). Budding efficiency was normalized to the Sec13/31A wt and delABD respectively. SEM of three independent experiments. **E.** Addition of excess (5 µg) ALG-2 inhibits vesicle budding independent of Sec31A.

To investigate whether the inhibitory effect of ALG-2 was mediated through an interaction with Sec31A, we mapped the ALG-2 interaction site on Sec31A using a peptide array (Figure 3A). The mapped ALG-2 target sequence (Figure 3B) corresponded to the ALG-2 binding site at amino acids 839–851 recently described by Shibata et al. [18]. Deletion of amino acids 827–852 of Sec31A led to abrogation of ALG-2 binding. The relative molar ratio of components collected using Ni beads and His-tagged Sec31A was estimated from fluorescence intensity readings to be 1∶1:2 for Sec13:Sec31A:ALG-2 for wild type Sec31A whereas only background levels of ALG-2 were seen in the case of Sec31A lacking the ALG-2 binding domain (Sec31AmutABD) (Figure 3C). Vesicle budding could be reconstituted when the wt recombinant Sec31A was substituted with the version lacking the ALG-2 binding domain indicating that the Sec31AdelABD is biologically active. Furthermore, we were able to reverse the inhibitory effect of ALG-2 by using the Sec31AdelABD in place of the wt Sec31A (Figure 3D) indicating that ALG-2 is acting directly through Sec31A to inhibit vesicle budding. We found that excess ALG-2 overcame the reversible budding inhibition of the Sec31AdelABD indicating a Sec31A-independent function for ALG-2 on the budded vesicles (Figure 3E). This could be due to an effect of ALG-2 on the fusogenicity of transport vesicles as described previously [5].

### ALG-2 Connects the Inner and Outer Coat of COPII in vitro

**Figure 4:**
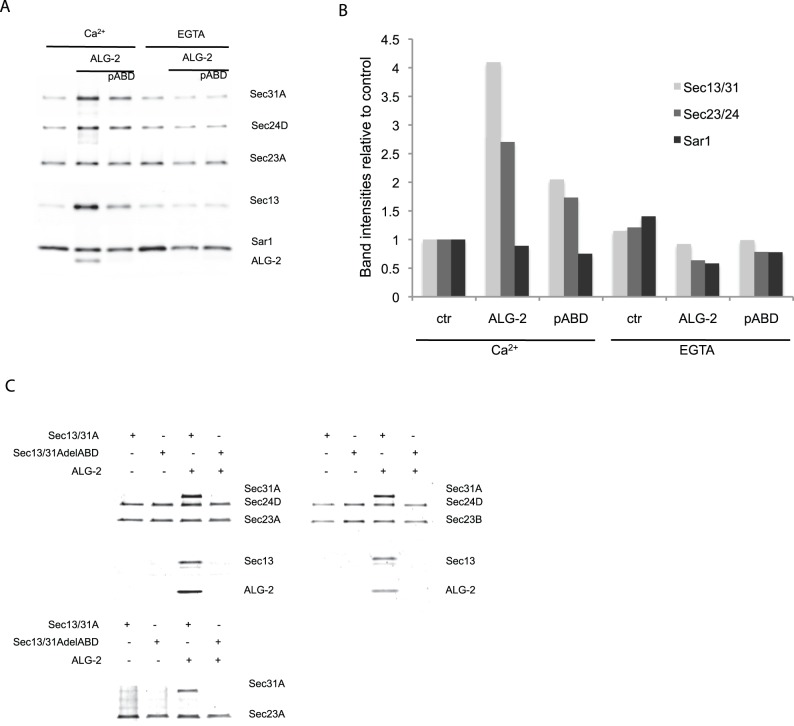
Sec31A is recruited to liposomes and Sec23 in an ALG-2-dependent manner. **A.** Recombinant proteins bound to artificial liposomes and collected from a sucrose gradient were analyzed for recruitment efficiency in the presence of ALG-2 and a peptide representing the ABD (pABD). Antibodies directed against Sec31A, HA tag (HA-Sec13), Flag-tag (flag-Sec23A), Sar1 and ALG-2 were used to analyze protein recruitment. The immunoblot is representative of more than three independent experiments. B. Quantification of band intensities relative to Ca^2+^ control (ctr) in representative immunoblot. **C.** Anti-Flag antibody coated beads were used as bait for flag-Sec23. Protein binding was performed in the presence of Ca^2+^ and proteins in gels were visualized using SyproRed staining.

In order to test whether ALG-2 was recruited along with the COPII complex on artificial liposomes [19], ALG-2 and COPII proteins were mixed with liposomes and then resolved by buoyant density gradient flotation. We found that in the presence of Ca^2+^ and ALG-2, both Sec23A/Sec24D and Sec13/31A were recruited to the liposomes to a higher extent than in the absence of ALG-2 (Figure 4A). Adding EGTA reversed this ALG-2-dependent recruitment and the recruitment was significantly diminished by the presence of a peptide representing the ALG-2 binding site of Sec31A, indicating that the recruitment of the COPII components was dependent on ALG-2 binding to Sec31A. Quantifying the protein band intensities relative to a 10–200 ng COPII recombinant protein standard (Figure S2), we found that the inner and outer coat proteins increased in the presence of ALG-2/Ca^2+^ but the levels of Sar1 appeared constant (Figure 4B) indicating that ALG-2 is capable of accumulating Sec23/24 and Sec13/31 to membranes irrespective of Sar1. To test whether ALG-2 could mediate binding of the outer COPII coat to the inner coat, we used flag-tagged Sec23A in complex with Sec24D as bait for the recruitment of Sec13/31A. Sec13/31A was recruited only in the presence of ALG-2/Ca^2+^ whereas the version of Sec31A with ABD deleted was not recruited. This was the case also for the Sec23B isoform in complex with Sec24D (Fig. 4C) as well as for Sec23A in complex with the Sec24A and Sec24C isoforms (not shown). Furthermore, we found that Sec23A itself was sufficient to recruit Sec13/31A in the presence of ALG-2/Ca^2+^. ALG-2 was only recruited in the presence of Sec31A suggesting that ALG-2 binding alters Sec31A to enable it to bind Sec23A rather than binding both Sec23 and Sec31A (Fig. 4C).

## Discussion

Though much is known about the molecules involved in transporting cargo from the ER to the Golgi (reviewed in [20], little is known about the role of possible cationic regulation of this process. We have identified ALG-2 and Ca^2+^ as novel regulators of the COPII budding process. ALG-2 inhibited the capture of membrane cargo proteins Sec22 and p58/ERGIC into vesicles in a cell-free COPII reaction, which reproduces the sorting of transport cargo that bud from the ER. This vesicle budding inhibition is mediated through an ALG-2/Ca^2+^ dependent binding of Sec31A. ALG-2/Ca^2+^ also mediates binding of Sec31A to Sec23 in the inner layer of the coat. This connection may interfere with cargo sorting by the Sec23/24 heterodimer.

The group of JC Hay previously described that ALG-2/Ca^2+^ inhibited heterotrimerization of a COPII membrane cargo protein, VSV-G protein, an assay they developed to quantify vesicle fusion [21]. They concluded that ALG-2/Ca^2+^ might block vesicle fusion to a target membrane by causing COPII to be retained on transport vesicles [5]. In our experiments ALG-2/Ca^2+^ inhibits budding though it may also block COPII vesicle targeting and fusion. Exceeding a 2∶1 molar ratio of ALG-2:Sec31A caused a Sec31A-independent block of budding, in that the Sec31AdelABD mutant did not restore budding inhibited by ALG-2 (Figure 3E). The Hay group found that the Ca^2+^-binding sites of ALG-2 were important for inhibition of fusogenicity, however, the molecular target of this ALG-2/Ca^2+^-dependent inhibition was not experimentally addressed [5]. In our experiments we have minimized the effect of both auxiliary factors in COPII budding as well as endogenous ALG-2 found in liver cytosol by reconstituting the COPII budding process with purified proteins. The membrane cargo protein p58/ERGIC was detected in vesicles produced in normal reactions but little or no cargo protein was detected in reactions containing ALG-2 (Figure 2E, Figure S1).

Previous work documented a Ca^2+^-dependent co-localization of ALG-2 with Sec31A possibly at the ER exit sites [1], [2], [3]. Depletion of ALG-2 in HeLa cells reduced the localization of Sec31A in membranes and correspondingly the depletion of Sec31A decreased ALG-2 in a membrane fraction [3]. The precise site of interaction of Sec31A with ALG-2 has not yet been identified. Based on crystal structure studies of ALG-2 it was suggested that a conformational change induced by Ca^2+^ binding would expose a hydrophobic crevice on the surface of ALG-2 by rotation of the masking an arginine (R125) allowing interaction with a peptide representing Alix/AIP1 [22]. Unlike Alix/AIP1, Sec31A binds to the alternative splice variant ALG-2.1 (Figure 2A) [23], [24]. This variant does not exert a switch mechanism for the corresponding R123 (reviewed in [25]), it is thus likely that the mode of ALG-2 binding to Sec31A differs from that of Alix as previously suggested [24]. Binding of Ca^2+^ to EF-hand 3 was described to cause an increase in hydrophobic residue exposure, whereas binding of Ca^2+^ to EF-hand 1 did not significantly change surface hydrophobicity [26]. Our findings show that binding of Ca^2+^ to EF hand 1 directs binding of ALG-2 to Sec31A (Figure 2A) and ALG-2/Ca^2+^-dependent vesicle budding inhibition. Yamasaki et al. showed that knockdown of Sec31A leads to removal of ALG-2 at the ER-exit site [3]. The retention kinetics of a fluorescently tagged version of Sec31A changed upon removing the ALG-2-binding site of Sec31A [18] indicating that ALG-2 affects the turn-over of Sec31A at the ER-exit sites. We were able to restore ALG-2/Ca^2+^ inhibited budding reactions on addition of a Ca^2+^ chelator, indicating that ALG-2 during Ca^2+^-transients attenuates vesicle budding. ALG-2 could be functioning as a safeguard during elevated cellular Ca^2+^ to ensure that COPII vesicles do not merge directly with the Golgi apparatus as proposed by Bentley et al. [5], but fuse to form ERGIC. Interestingly ALG-2 is conserved in higher eukaryotes (reviewed in [27]) whereas yeast, which are believed to be void of ERGIC, has a common ancestral penta-EF hand (PEF) family protein. It is possible that ALG-2 has evolved in higher eukaryotes in order to regulate sorting of proteins to ERGIC. The PEF-family yeast homologue, Pef1p was recently reported to bind to the Sec13/31p in a Ca2+-independent manner [28]. We were not able to detect binding between Sec31A and the mammalian homologues of Pef1p, Peflin (not shown). We propose that the attenuation of the COPII budding process facilitates transport of specific cargo in a Ca^2+^-dependent manner. Perhaps ALG-2 acts to stall the budding process to synchronize cargo sorting and vesicle morphogenesis. A role in cargo-dependent regulation of export processes was recently discoverered for Sedlin, a member of the TRAPP complex. Sedlin knockdown was shown to specifically decrease procollagen packaging whereas neither secretion of other tested proteins nor the trafficking of VSV-G was affected. Sedlin directly interacted with Sar1 preferentially in its GTP bound state presumably regulating Sar1 cycling at ER exit sites and thereby the stability of membrane buds [29]. It is possible that ALG-2 exerts a similar effect through Sec31A by regulating the availability of Sec13/31A for the Sec31A/Sec23 interaction. Previously it was shown that addition of cytosol enhanced the recruitment of COPII proteins to membranes indicating that cytosolic factors assist coat subunit recruitment or coat assembly [15]. We find that ALG-2 facilitates the binding of Sec13/31A and Sec23, although previous reports have shown that Sar1 in concert with Sec23 is needed for interaction with Sec31 [30], [31] and more specifically bind an active fragment of Sec31A, which stimulates the GAP activity of Sec23 [31]. Both the ALG-2 binding site and the active fragment of Sec31A are situated in the proline-rich region of Sec31 and it is possible that ALG-2 modulates this domain to accommodate the Sec31A binding to Sec23. Perhaps ALG-2 replaces the bridging effect of the active fragment and by this inhibits GAP activity and vesicle release.

It is possible Ca^2+^/ALG-2 signals a cellular requirement to arrest cargo transport vesicle budding during periods of ER stress or other cellular processes where a protein export halt would be advantageous. The recent discovery that monoubiquitination of Sec31A is essential for collagen secretion in mouse embryonic stem cells [14] opens for novel experimental approaches in concert with the present discovery of an ALG-2/Ca^2+^-dependent attenuation of vesicle budding. Furthermore, Sec31A phosphorylation and membrane association was found to be regulated by casein kinase 2, which also affected ER to Golgi traffic [32]. Future work in this field will be directed at investigating whether ALG-2/Ca^2+^ contributes to regulation of the formation of large COPII vesicles through these postranslational modification processes. Aberrant trafficking and enhanced surface expression of the T-cell receptor could explain the apparent role of ALG-2 in promoting T-cell receptor-mediated cell death [6]. Based on our previous research we do not find a direct function of ALG-2 in apoptosis, rather a necessity for ALG-2 in cell proliferation [33], [34]. It could be that the correct expression at the cell surface of certain growth regulators depends on ALG-2 through its effect on ER to Golgi trafficking. Experiments investigating the plasma membrane proteome following elevated Ca^2+^-levels in the presence and absence of ALG-2 will be important to further study the role of ALG-2/Ca^2+^ in protein transport.

## Materials and Methods

### Buffers

Phosphate-buffered saline without calcium and magnesium (PBS, pH 7.4) was purchased from Mediatech (Herndon, VA). TBS contains 50 mM Tris-HCl (pH 7.4), 150 mM NaCl. TBST is TBS supplemented with 0.1% (w/v) Tween 20. Buffer A (budding): 20 mM HEPES-KOH (pH 7.4), 250 mM sorbitol, 150 mM potassium acetate; buffer B (protein purification): 20 mM HEPES-KOH (pH 8.0), 10% (w/v) glycerol, 250 mM sorbitol, 500 mM potassium acetate, 0.1 mM EGTA, 5 mM ß-mercaptoethanol, 10 mM imidazole; buffer C (protein purification): 20 mM HEPES-KOH (pH 8.0), 10% (w/v) glycerol, 250 mM sorbitol, 0.1 mM EGTA, 5 mM ß-mercaptoethanol, 10 mM imidazole; buffer D (protein purification): Buffer B with 50 mM imidazole; buffer E (protein purification): Buffer B with 250 mM imidazole; buffer F (Liposome flotation): 20 mM HEPES-KOH (pH 6.8), 110 mM potassium acetate, and 2 mM MgCl_2_; buffer G (M2 immunoprecipitation): Buffer A with 0.1% NP40 and 100 µM CaCl_2_. Buffer H consists of 150 mM Tris-HCl (pH 6.8), 15% (w/v) SDS, 25% (w/v) glycerol, 0.02% (w/v) bromphenol blue, and 12.5% (w/v) 2-mercaptoethanol. Buffers A–H were added EDTA-free protease inhibitor mix from Roche (Roche).

### Mapping of Sec31A ALG-2-binding Site

The ALG-2 binding sequence of human Alix/AIP1 was used in silico to find the most probable binding motif of Sec31A. Overlapping peptides (20-mer) representing amino acids 808–869 were synthesized on a nitrocellulose membrane as previously described [35] and overlaid with fluorescein labeled recombinant ALG-2 (0.2 µg/ml) in a buffer containing 50 mM Tris-HCl, pH 7.5, 0.5% BSA, 0.05% Tween 20, 0.15 M NaCl, 0.3 M arginine and either 1 mM CaCl2 or 5 mM EDTA. Spot intensities were measured using a Typhoon scanner.

### Construction of the ALG-2-binding Deficient Sec31A

Deletion of the ALG-2 binding site corresponding to amino acids 827–852 of human Sec31A was carried out using forward and reverse primers 23 bp upstream and downstream of the deleted sequence. Two PCR reactions were carried out in parallel, one with the forward primer in combination with a primer containing a unique Eco47III site in the Sec31 sequence and the forward primer with a unique Spe1 site in the plasmid sequence. The PCR products were mixed and used as primers and templates in a second PCR reaction. The PCR product was then cut with the two enzymes and cloned back into the vector. Sequencing confirmed the correct deletion.

### Protein Purification

Recombinant ALG-2 was purified as previously described [16]. Mammalian COPII proteins were inserted in the Bac-to-Bac baculovirus expression system (Invitrogen) and purified in accordance with procedures described in [36]. In brief pHA-Sec23A/B, and pHis-Sec24A/B/C/D were co-expressed as was pHA-Sec13 and either pHis-Sec31A or pHis-Sec31ADelABD in ES-SF9 cells. Human GST-Sar1 WT and H79G were purified from *Escherichia coli* as described [15]. FLAG-Sec23/His-Sec24 and HA-Sec13/His-Sec31 complexes were purified using Ni-NTA affinity chromatography as described in [36].

### In vitro Vesicle Budding Assay

The vesicle budding assay was performed as described previously [37]. In brief, HeLa cells were harvested in PBS and permeabilized by 5 min incubation in Buffer F containing 40 µg/ml digitonin. Permeabilized cells were washed in Buffer A and used at 40 ug protein/reaction. The budding reaction was assembled using either rat liver cytosol at 4 mg/ml or purified Sar1A/B, HA-Sec13/His-Sec31A and Flag-Sec23A/His-Sec24D. Unless otherwise stated, an ATP-regenerating system was added resulting in final concentrations of 1 mM ATP, 40 mM creatine phosphate, 0.2 mg/ml creatine phosphokinase and 0.1 mM GTP. Unless otherwise stated, 0.3 µg rALG-2 was added to reactions when indicated. Budding reactions were assembled on ice and incubated for 30 min at 30°C then put on ice. The donor membranes were removed by a 20 min 12,000×g centrifugation and the supernatant fraction was further centrifuged for 25 min at 55,000 rpm at 4°C using a TLA100 rotor in a Beckman Optima TLX ultracentrifuge to sediment the vesicle products. To fractionate the budding vesicles by flotation, we mixed the budding reaction 1∶1 with 80% (w/v) Nycodenz (Progen) in buffer A. The reaction was overlaid with a gradient of 35-10% Nycodenz in buffer A and centrifuged for 2 h at 55,000 rpm in a TLS-55 rotor. Fractions were collected from the top and mixed with Buffer H for separation by SDS-PAGE and immunoblot analysis.

### Liposome Flotation Assay

Detailed procedures for the liposome-binding assay were described in Matsuoka and Schekman [38]. The liposomes were prepared from a liposome mix containing 31.8 mM dioleoylphosphatidylcholine, 33.6 mM dioleoylphosphatidylethanolamine, 12.3 mM dioleoylphosphatidylserine, 13.9 mM dioleoylphosphatidic acid, 11.7 mM phosphotidylinositol, 5.0 mM phosphotidylinositol phosphate, 4.6 mM phosphotidylinositol bisphosphate, 4.8 mM cytidine diphosphate diacylglycerol, 0.7 mM Texas Red phosphotidylethanolamine (TX-PE, Molecular probes). A liposome suspension (20 µl) was mixed with 1.6 µg of Sar1A, 1.7 µg of Sec23A/24D, 2 µg of Sec13/31A, 100 µM GTPγS and up to 80 µl of HKM buffer was added following addition of ALG-2, pSec31AdelABD, Ca^2+^ and EGTA as stated in Figure 4. Binding was performed for 30 min at 30°C and the liposome/protein solution was mixed with 50 µl 2.5 M sucrose in HKM buffer followed by overlaying first with 100 µl 0.75 M sucrose in HKM and then with 20 µl HKM. Following a 20 min centrifugation at 100,000 rpm using a TLA100 rotor, the top fraction was collected and loaded on a gel normalized to TX-PE fluorescence.

### Sec23 Binding

Anti-Flag M2 (Sigma-Aldrich) affinity gel was washed twice with Buffer G and 4 µg Flag-Sec23/His-Sec24 complexes per reaction were immobilized on the matrix and incubated for 20 min at 4°C. Sec13/31A (5 µg) or Sec13/31AdelABD were added to the matrix along with 0.3 µg rALG-2. Proteins were incubated with matrix for at least 2 h at 4°C before three washes of matrix in Buffer G. Bound proteins were eluted using 0.25 mg/ml Flag peptide in Buffer G.

### Antibodies

Polyclonal antibodies against ALG-2 were as previously described [39]. Monoclonal antibodies against the His-tag (Penta-His) and Sec31A (clone 32) were purchased from Qiagen and BD Biosciences, respectively. Detection by immunoblot analysis using polyclonal anti-ERGIC-53, anti-Ribophorin-I, anti-Sec22b, and Sar1 rabbit antiserum was performed as previously described [37].

### Protein Visualization

Proteins were resolved on 10% or 4–20% gradient SDS gels and either transferred to PVDF membranes by wet electroblotting followed by visualization using the LI-COR infared imaging system (Li-cor Biosciences) or stained with SYPROred (1∶5000, Invitrogen) in 7.5% acetic acid and scanned in a Typhoon (GE Life Sciences). Quantification of protein intensities was performed using either the Odyssey or ImageJ image analysis software.

## Supporting Information

Figure S1 **Buoyant budded vesicles containing p58/ERGIC do not contain ALG-2.** The product of vesicle budding reactions performed with or without recombinant ALG-2 was resolved in a 10–40% Nycodenz gradient and fractions were analyzed for the presence of p58, Sec31A and ALG-2. The right hand side input lanes were run on separate gels.
(EPS)

Figure S2 **Titration of recombinant COPII proteins for quantification of liposome binding.** 10–200 ng of recombinant proteins were analyzed following SDS-PAGE and Western blot analysis using the antibodies described in materials and methods.
(EPS)
